# Challenges of SARS-CoV-2 genomic surveillance in India during low positivity rate scenario

**DOI:** 10.3389/fpubh.2023.1117602

**Published:** 2023-06-27

**Authors:** Siddharth Singh Tomar, Krishna Khairnar

**Affiliations:** ^1^Environmental Virology Cell (EVC), Council of Scientific and Industrial Research-National Environmental Engineering Research Institute (CSIR-NEERI), Nagpur, India; ^2^Academy of Scientific and Innovative Research (AcSIR), New Delhi, India

**Keywords:** pandemic (COVID-19), SARS-CoV-2, genomic surveilance, whole genome sequencing, public health

## Abstract

Being the second most populous country in the world, India presents valuable lessons for the world about dealing with the SARS-CoV-2 pandemic. From this perspective, we attempted a retrospective evaluation of India’s SARS-CoV-2 genomic surveillance strategy and also gave some recommendations for undertaking effective genomic surveillance. The dynamics of the COVID-19 pandemic are continuously evolving, and there is a dire need to modulate the genomic surveillance strategy accordingly. The pandemic is now settling towards a low positivity rate scenario, so it is required to revise the practices and policies formulated for a high positivity rate scenario. The perspective also recommends adopting a decentralised approach for SARS-CoV-2 genomic surveillance with a focus on optimising the workflow of SARS-CoV-2 genomic surveillance to ensure early detection of emerging variants, especially in the low positivity rate scenario. The perspective emphasises a key observation that the SARS-CoV-2 genomic surveillance is an important mitigation effort during the pandemic, the guards of such mitigation efforts should not be lowered during the low positivity rate scenario. We attempt to highlight the limitations faced by the Indian healthcare administration during the SARS-CoV-2 genomic surveillance and, simultaneously, suggest policy interventions derived from our first-hand experience, which may be implementable in a vast, populated country like India.

## Introduction

Since the first outbreak was reported in December 2019, the COVID-19 pandemic is now running in its third year. The human and financial loss of this pandemic is staggering. The low and middle-income countries (LMICs) are on the list of regions worst affected by this pandemic. As per the lancet commission report of 2022 (1). The failure in international cooperation and lack of support to these countries aggravated the damage as the pandemic unfolded. This report also highlights the lack of timely and accurate data sharing on emerging variants during subsequent waves. The scientific community was uncertain about the kinetics of the virus at the onset of the pandemic. In the earlier phase of the pandemic, the major focus was on testing, contact tracing, and isolation of positive cases. India was one of the first countries which impose a nationwide lockdown (2); similar restrictions were enforced across the globe, impacting not only the Indian economy but also the world economy. Subsequent waves of the COVID-19 virus revealed that the virus could mutate into different variants, as was the case with the delta variant, which emerged from India (3) and caused the deadly second wave worldwide. The two primary COVID-19 testing methods, antigen detection, and Reverse Transcriptase Polymerase Chain Reaction (RTPCR) tests were deployed globally, with the RT-PCR being the gold standard for the detection of positive cases (4). With the progression of the pandemic, it was evident that only testing and isolation of positive COVID-19 patients was not enough to mitigate the pandemic; there was also a dire need for early detection of mutations taking place in the genome of the SARS-CoV-2 virus to understand the dynamics of emerging variants (5). The detection of SARS-CoV-2 variants is only possible through whole genome sequencing (WGS) of the viral genome (6). This warranted a mammoth task of establishing a nationwide genome sequencing infrastructure, which is at par with the international practices of genomic surveillance. Hence, along the lines of the COVID-19 Genomics United Kingdom Consortium (COG-UK) (7); on 30th December 2020, India also came up with the Indian SARS-CoV-2 Genomics Consortium (INSACOG) (8). The mandate of this multi-agency consortium includes ascertaining the prevalence of variants of interest (VOI) and variants of concern (VOC) in the population. As per INSACOG recommendations, it is mandated to sequence 3–5% of RT-PCR-positive COVID-19 samples in the country (9). The number of SARS-CoV-2 positive samples is usually high during the peak of the pandemic, so there was a need for criteria for screening the samples for sequencing; therefore, INSACOG proposed inclusion criteria for WGS of SARS-CoV-2 positive samples, preferably having a Cycle Threshold (Ct) values ≤25 (9). We believe that this strategy may work well in a scenario with a high positivity rate (HPR), as a large number of positive samples are available with a high viral load (Ct value ≤25). However, the dynamics of WGS may get affected adversely when the positivity rate declines and gradually shifts towards scenarios of moderate positivity rate (MPR) and low positivity rates (LPR). In LPR-MPR scenarios, there is a drastic reduction in the number of qualified positive samples with Ct values ≤25 for WGS, this may cause a drastic fall in the overall sequencing rate due to a prolonged waiting period, leading to adversely impacting the chances of early detection of any potential VOI/VOC. The present criteria of Ct value ≤25 also increases the risk of missing vaccine breakthrough events and re-infection cases. Let us assume that a sample ≥25 Ct for a vaccine breakthrough event may escape the genomic surveillance by WGS. To overcome this limitation, it is proposed to use customised genome sequencing workflows for each of the three scenarios, like HPR, MPR, and LPR, to maintain an optimum WGS rate.

### Genomic surveillance strategy of India

SARS-CoV-2 genomic surveillance in India is designed on the lines of COG-United Kingdom (7) and on the recommendations of WHO for genomic surveillance (10). The effort is coordinated at the national level by INSACOG through a 3 tier organisational structure; that includes a nodal unit at the national centre for disease control (NCDC) (11). There are 10 INSACOG genome sequencing laboratories (IGSLs); these hub IGSLs are associated with 46 satellite labs (Satellite IGSLs) for sequencing (12). The sequencing workflow primarily depends on Sentinel Sites identified by respective states for a sample collection from primary centres. The INSACOG recommends ensuring that at least 80% of districts of every state are represented. Sentinel sites are expected to send a minimum of 15 samples per 15 days to the IGSL; this criteria of 15 samples could be relaxed if the daily reported cases at the sentinel site are less than one. It is recommended that from the sentinel sites, the RTPCR-positive samples are transported to the IGSLs in the cold chain. IGSL will conduct the sequencing and share the data on INSACOG and IHIP portals (12). LPR conditions could lead to a decrease in the sample inflow from sentinel sites. To maintain a healthy sequencing number, it is recommended by the WHO to include Non-Sentinel labs, including non-government (privately owned labs), in the genomic surveillance network (13). It is evident that the genomic surveillance system in India is highly centralised. We propose that the LPR scenario is an ideal stage to decentralise this system and allow the state governments and local health administration to plan and execute genomic surveillance studies at the local level, given local healthcare requirements and resource availability. Genomic surveillance in a decentralised system will have better demographic representation and broader geographical coverage. WHO has also endorsed the idea of local-level planning in its global genomic surveillance strategy (14). Objective three of this strategy indicates facilitating the implementation of genomic surveillance from the local to the global level and to build the capacity for adopting genomic surveillance as a regular public health practice. Early action at the local level will prevent the broader spread of VOCs and will help in preventing the human and capital loss associated with the pandemic (14).

### A need to review: Ct value-based selection criteria for SARS-CoV-2 genomic surveillance in India

INSACOG recommends that “Only those samples which are positive for SARS-CoV-2 by RT PCR preferably with the Ct value of 25 or less should be packaged and transported” for WGS.9 The possible reason for using the criteria of ≤25 Ct could be to avoid sequencing failures, considering the SARS-CoV-2 RNA content in the samples. However, the samples with >25 Ct could also be used for sequencing to generate lineage/variant-level results (13). The existing criterion is perhaps designed to fit an HPR scenario but not for an LPR scenario, where the positive samples have low SARS-CoV-2 RNA content. Therefore, there is a need to revise the selection criteria of <25 Ct as there is a looming risk of missing the emerging variants during the LPR scenario. An interesting fact also resonates with this viewpoint that the Ct value in a positive sample may correlate with the viral RNA content in the collected sample but may not be an accurate representation of the viral load in the infected individual (15). It is essential to understand that the oropharynx (OS), nasopharynx (NS), and anterior nares (AN) are the preferred collection sites for RT-PCR and antigen detection (16); these sites are initially targeted by the SARS-CoV-2 virus only for acclimatisation and localisation, before progressing towards the main target site like lungs. Hence, the testing done from samples collected from OS/NS/AN is less sensitive in detecting the SARS-CoV-2 virus in the initial and late presentation of the disease (17). Although the diagnosis of the SARS-CoV-2 virus in the OS/NS/AN sites comes in handy for a high-throughput diagnosis, once the virus has progressed to the main target site and has reduced predisposition in the OS/NS/AN, there is an ambiguity over the actual viral load detected by the testing methods (18). The Ct value, a quantitative representation of Viral RNA load in the given sample, depends on various factors such as stage of Infection, virus localisation, sampling technique, transportation conditions, sample handling, and RNA extraction. Ct value is dependent on the amplification of Viral RNA during the kinetics of a PCR reaction; mutation in the primer binding region could result in a poor primer binding with the template strand; this poor primer binding could disrupt the PCR kinetics and may result in higher Ct values even in high viral load samples (19). Studies also showed that some variants of the SARS-CoV-2 virus-like Omicron and B.1.616 are poorly detected in some RT-PCR tests; these variants possess mutations in the S gene; the RTPCR fails to amplify the S gene in the sample leading to poor detection (20, 21). Therefore, applying the <25 Ct value criteria for SARS-CoV-2 WGS may not be a foolproof approach, this may also negatively impact the purpose of random sample selection for SARS-CoV-2 sequencing.

### Sample size and sample representation for considerations at different prevalence levels

**Figure 1:**
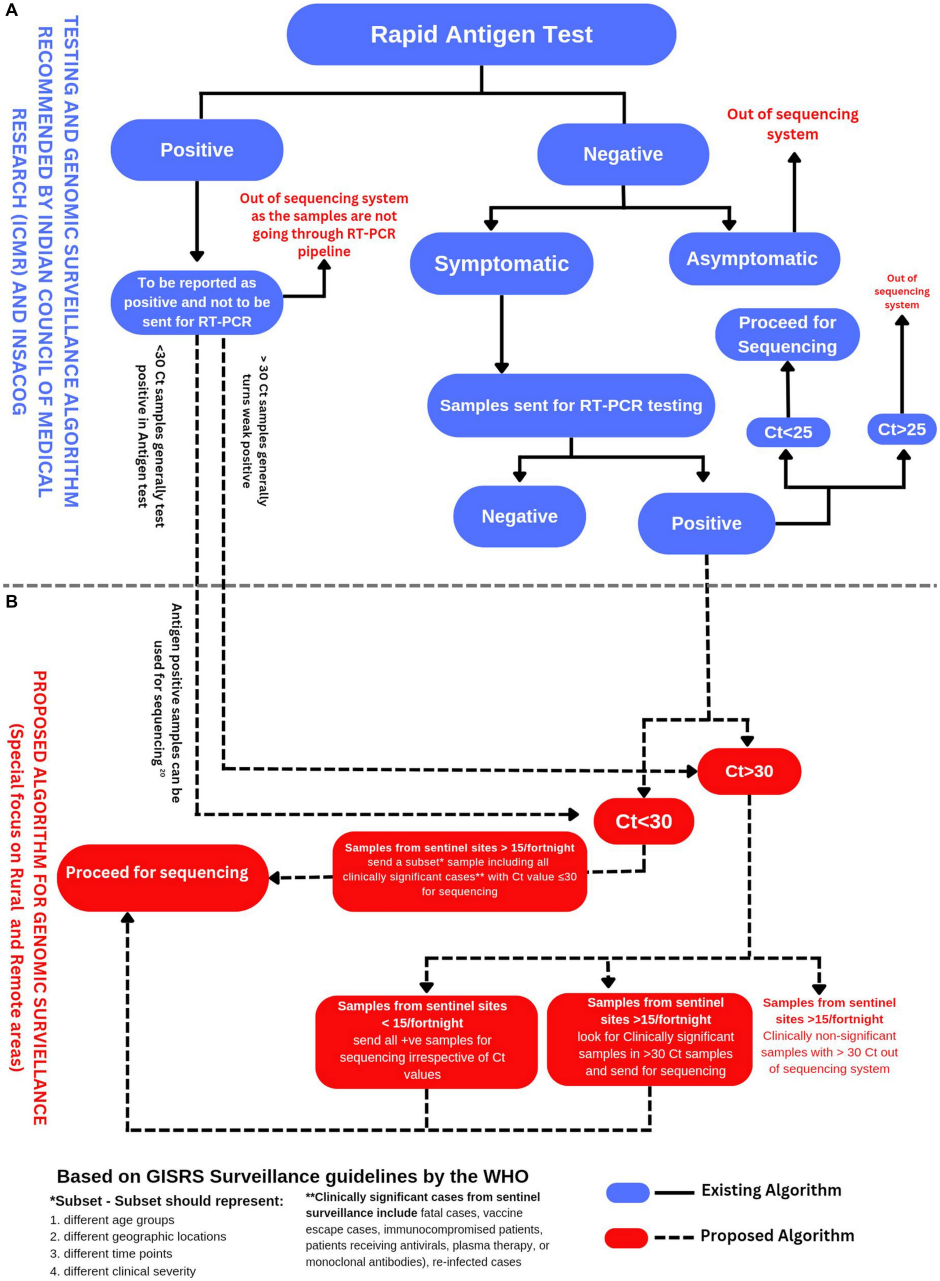
**(A)** Testing and genomic surveillance algorithm recommended by ICMR (23) and INSACOG (9). **(B)** Proposed algorithm for genomic surveillance with a particular focus on rural and remote areas of India based on the recommendations of WHO in end-to-end integration of SARS-CoV-2 and influenza sentinel surveillance (13).

The sample size for SARS-CoV-2 genomic surveillance depends on two factors, the number of total positive cases and the level of prevalence of specific variants. The European Center for Disease Control (ECDC) proposed sampling guidelines concerning both parameters (22). For example, to detect a variant with 1% relative prevalence in a total positive sample range of 50,001–100,000, it is necessary to sequence at least 1,500 samples per week to get a 95% confidence result. However, to get similar confidence results in the same positive sample range of 50,001–100,000 for a variant with 5% relative prevalence, it is necessary to sequence only 292 samples per week. These recommendations clearly emphasize keeping the sampling strategies in sync with the total no. of positive cases and prevalence levels of the variants. This information also implies that if we sequence fewer samples in LPR-MPR scenarios, there are greater chances for new emerging variants with low initial prevalence to escape the net of sequencing surveillance. In the Indian scenario, we observe that after the decline of the SARS-CoV-2 Delta wave, there is a predominance of Omicron. Since January 2022, the Omicron variant of SARS-CoV-2 has been prevailing in an LPR-MPR scenario. It is interesting to note that Omicron has not yet given rise to an HPR scenario, but at the same time, Omicron continues to persist chronically and mutate further. We believe that the chronic persistence of the Omicron variant over the last 10 months could be attributed to the lacunas in the workflows of the COVID-19 policy of genomic surveillance. Perhaps, the policymakers failed to readjust the yardsticks promptly concerning the changing situation from an HPR to an LPR scenario, especially in the case of Omicron. We have made a schematic representation of the existing workflows and the proposed workflows for SARS-CoV-2 genomic surveillance, with particular emphasis on ramping up the sequencing efforts in the LPR scenario (Figure 1).

## Genomic surveillance should be viewed as an early warning system

**Figure 2:**
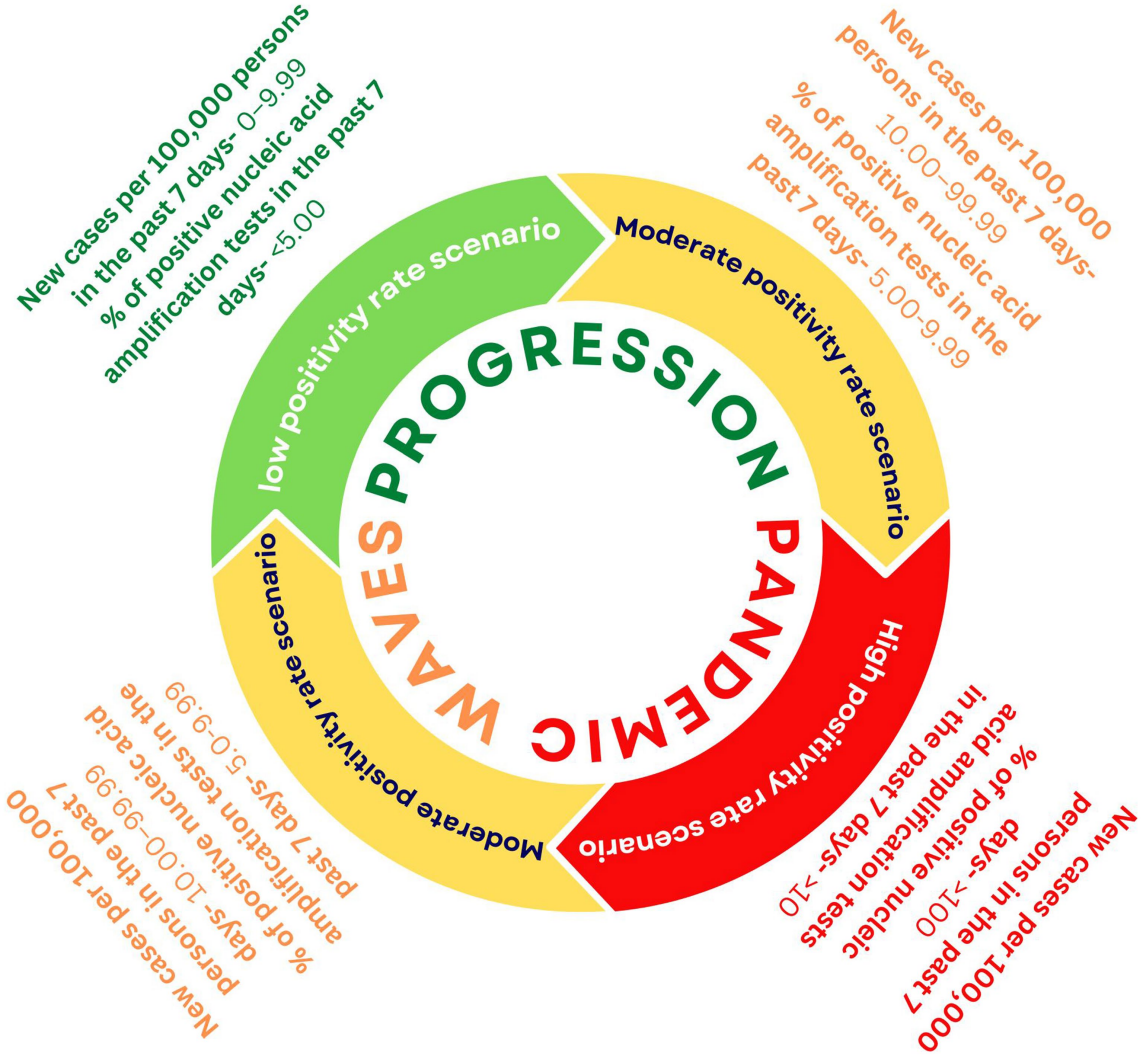
Cyclical progression of the pandemic and proposed positivity rate thresholds for the Indian scenario as per CDC criteria (24).

The pandemic progresses in a cyclical process amongst three scenarios starting with an LPR, then moving towards an MPR, and finally to an HPR (Figure 2). To better manage the pandemic, we need customised strategies for each of these scenarios. For this, we need to amend the existing SARS-CoV-2 genomic surveillance policy, which tackles the issue with the approach of ‘one size fits all’. India is currently using a fixed sampling strategy from sentinel sites. However, this approach is operationally practical but has an under-representation of the population and low chances of variant identification at the early stages of the pandemic wave. As mentioned in the interim guidelines for genomic surveillance of the SARS-CoV-2 pandemic issued by the WHO in August 2021, a variant circulating among the population at lower levels, such as 1%, will require a larger sample size when compared to the variant circulating at a higher level of prevalence (10). As per the latest GISAID data accessed on October 2022, India has sequenced only 0.5% of its total SARS-CoV-2 cases with an average turnaround time of 58 days for reporting the sequences (25). The target for SARS-CoV-2 WGS, as set by INSACOG, is 3–5% of total positive cases. Therefore, we propose that the focus should be on ramping up the genomic surveillance efforts and also using SARS-CoV-2 genomic surveillance as an early warning system (26), especially during the LPR stage of the Pandemic.

## The pandemic is not a monolith

When we look at the SARS-CoV-2 pandemic, we either look at it as a single event or as a combination of subsequent waves, this perspective can not do justice to the intricacies of the pandemic progression. There is a period of LPR between two pandemic waves, mathematical models suggest that this period is suitable for executing aggressive interventions to avoid or delay the onset of upcoming waves (27). Distributing each wave into three scenarios, HPR, MPR, and LPR, will aid in planning the mitigation strategy more effectively. The methods for sample selection, sample collection, sample transportation, Nucleic acid extraction, molecular testing, WGS platforms, and bioinformatic analysis can be modulated for each scenario.

One size does not fit all! The role of WGS in an HPR scenario is more likely to be quantitative and less likely to be qualitative. During an HPR scenario, a single VOC typically dominates or overwhelms the distribution (28). The WGS efforts during the HPR scenario would quantify the dominant variants. However, during the LPR scenario, the role of sequencing is more likely to be qualitative than quantitative alone. The sequencing data would not only identify different VOCs/VOIs but also give a clear picture of their relative distribution amongst total WGS samples. In other words, we can say that the chances of early identification of a new or emerging variant are relatively higher in a scenario. Arguably, the sequencing rates are considerably low during the scenario, which indicates that at the policy level, our guards are being lowered.

## Importance of defining positivity rates: low, moderate, and high

Before using the criteria of LPR, MPR, and HPR scenarios as a reference for making public health decisions, it is essential to define these terms first clearly. The United States Center for Disease Control (CDC) has defined these scenarios based on positivity rate (Samples tested positive/total samples tested × 100) as Low transmission- <5.0%, Moderate transmission- 5.0-7.9%, Substantial transmission- 8.0-9.9%; and High Transmission ≥10.0% (24). We recommend using the positivity rate as an indicator to plan the genomic surveillance in India by further simplifying CDC’s criteria by combining the categories of Moderate and Substantial transmission as “MPR” with an associated positivity rate range of 5–9.9%. Combining Moderate and Substantial transmission categories will ensure higher levels of preparedness and increased genomic surveillance for an extended period. Therefore, we propose three scenarios for guiding SARS-CoV-2 genomic surveillance in the Indian context: LPR (<5%), MPR (5–9.9%), and HPR (≥10.0%; Figure 2). However, this metric of positivity rate should be considered with caution as it may be prone to inaccuracies and could give an incomplete picture of the prevalence of the virus in the population, as the positivity rate highly depends on the number of individuals being tested. While considering the positivity rate metric, the Public health authorities must also consider factors like the severity of the disease, vaccine escape instances, reinfection cases, and fatality rate.

## Perspective of selection pressure on SARS-CoV-2

It is observed that during an HPR scenario, usually, a single variant dominates amongst a large population carrying a new wave of the pandemic (28). However, the selection pressure is also exerted on the virus through cohort immunity progressively acquired by natural infection and vaccination, which leads to a gradual decline in the number of cases after hitting the peak. It will be prudent to look for the emergence of new variants in this declining phase, as selection pressure would create a contrast between a declining variant and an emerging variant. To better understand this, let us take the example of mutations in the Receptor Binding Motif (RBM). RBM is a part of the Receptor Binding Domain (RBD) located within the Spike protein gene (S gene) of the SARS-CoV-2 genome. RBM is associated with the functions of viral entry as it facilitates the binding of RBD with hACE2 receptors. SARS-CoV-2 neutralising antibodies target the RBM. A study by Thompson and colleagues showed that RBM is a variable region prone to mutations, with the potential to create immune escape variants (29). There are also some non-RBD regions on the spike protein that can contribute towards increasing the infectivity (30). Such as the N-terminal domain (NTD) on the S-1 subunit of the spike protein. This region is involved in facilitating the attachment of the virus with the hACE2 receptor. NTD also serves as an attachment site for monoclonal antibodies (31). Mutations at NTD may increase the infectivity and immune evasion of the SARS-CoV-2 virus (32). Computational analysis showed that the b9-b410 and b14-b15 loops of NTD are important for antibody attachment, while the b14-b15 loop is critical for antigenicity. Mutations in these loops may affect immune escape in SARS-CoV-2 variants (33). It is observed that multiple SARS-CoV-2 variants, including B.1.1.7, B.1.351, and P1, have frequent mutations at the NTD, indicating continuous selection pressure favouring the mutants, and the need to develop NTD-specific neutralising mAbs for protective immunity and vaccine design (34).

The immune escape variants are more likely to emerge once the previous peak of infection has subsided due to cohort immunity. It is important to understand that the selection pressure acts like a filter, which will allow the immune escape variants to become dominant. The immune escape variant emerges in the LPR scenario and becomes a dominant variant in the HPR scenario. Hence, at the stage of an LPR scenario, the early warning signs can be appreciated for an emerging variant by the surveillance team to alarm the policymakers for effectively managing the pandemic.

**Figure 3:**
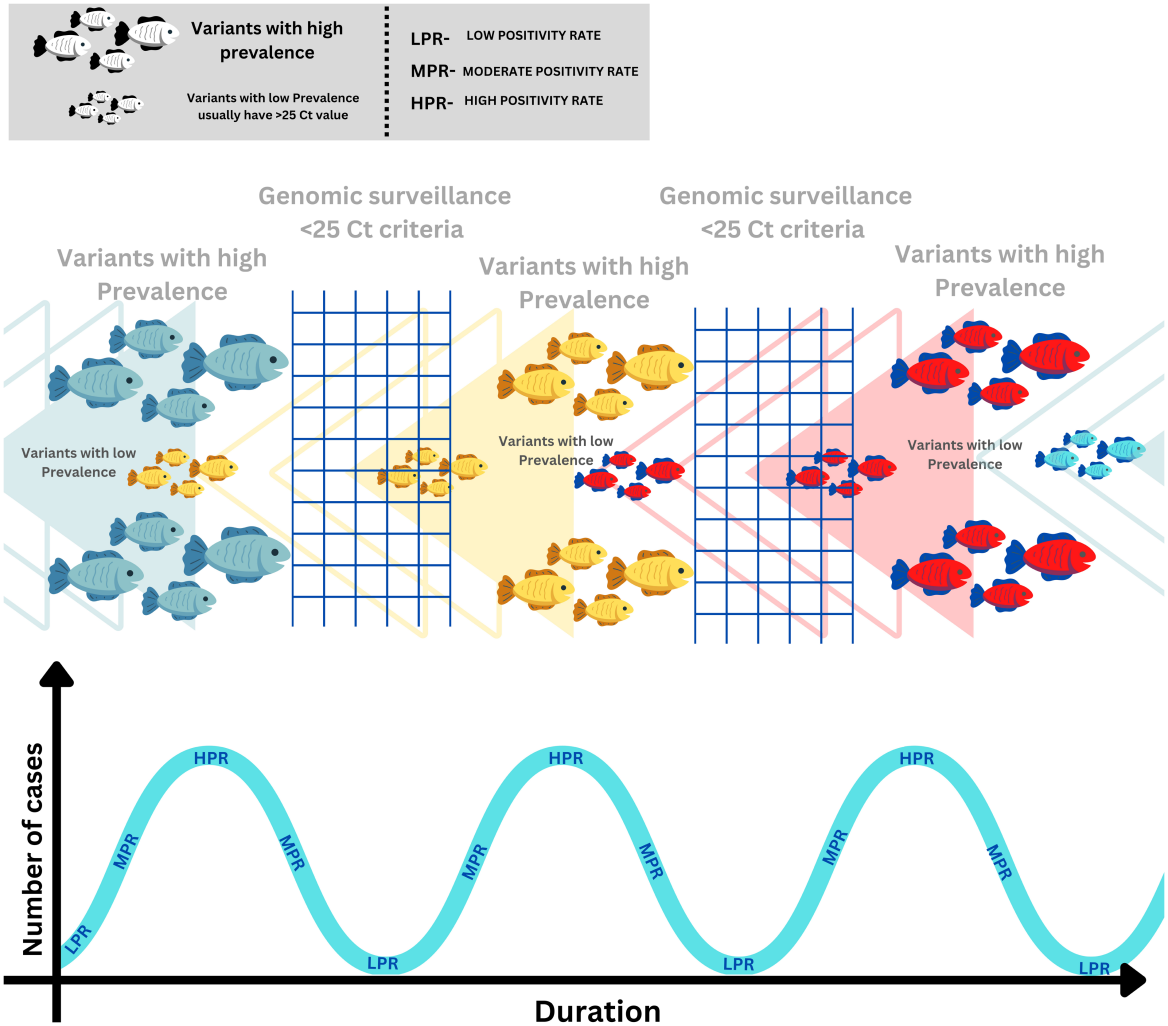
Schematic representation of SARS-Cov-2 variant dynamics in correlation with SARS-CoV-2 genomic surveillance.

Let us try to understand this from the point of view of a fishing net analogy, assuming that a fishing net is designed to catch a fish of large size. When large fishes are abundant in the water, the net will work selectively, but when the large fishes are not available in the water, and only small fishes are left, such fishing nets will be of no use. The fishermen need a fishing net with smaller netting to catch smaller fish. Similarly, when the HPR scenario is over, and the dynamics of the pandemic are shifted towards an LPR scenario, we need to adapt to a different approach that is more suitable for detecting emerging variants, especially in LPR scenarios. The early detection of these emerging variants in the LPR scenario will significantly help control the pandemic. Unfortunately, it is the declining phase of the pandemic where the guards are put down at the policy level, which needs to change considering the concurrent waves of the pandemic. A schematic representation using a fishing net analogy for explaining the dynamics of the escaping variants during a pandemic wave due to existing SARS-CoV-2 WGS criteria is delineated in Figure 3.

## Actionable recommendations

### Sample type and sample collection

To increase the efficacy of the SARS-CoV-2 genomic surveillance workflow, there is a need to address lacunae present at every stage. Collecting samples is a crucial step in the workflow as it has an overall impact on the sequencing success. Sample collection for SARS-CoV-2 could be classified as Upper respiratory tract (URT) and Lower respiratory tract (LRT) sampling. Methods such as Mid Turbinate swabs (MTS) (35), OS/NS Swabs in Virus Transport Medium (VTM) (36), OS/NS dry Swabs (37), Gargle method (38–40), and Anterior Nares collection (41) come under URT sampling, while more invasive methods like Endotracheal aspirate (ETA) (42) Broncho-Alveolar- lavage (BAL) (43) comes under the LRT sampling. These sample collection methods have advantages and disadvantages, which must be considered when opting for them. A study comparing the URT and LRT techniques showed that the LRT techniques are more sensitive and accurate in detecting infection. However, the LRT sampling techniques are deeply invasive, time-consuming, cumbersome, not high-throughput, and not used widely for testing a large population. A study comparing the efficacy of different URT sampling techniques showed that the mid-turbinate and NS swabs were more sensitive in detecting infection when compared to the anterior nares and OS swabs (44). Studies also revealed that the availability of viruses in these samples is time-dependent; it was observed that the probability of getting a false negative result is more during the initial and late presentation of the disease, indicating a detection window period (17). It is also observed that the healthcare workers involved in the NS/OS sampling for SARS-Cov-2 may wrongly perceive the location of sampling because of inadequate training; in such situations, the technician may misjudge that the path from the nostril to the nasopharynx through the dorsum of the nose, the ideal path of sampling is along the bottom of the nose facing backward toward the ear (44). An improper NS sampling might reduce the procedure to a ‘nasal swab’ rather than a proper ‘Nasopharyngeal swab’. The NS technique, when correctly done, is painful to experience, and a patient or the technician may retract the swab prematurely even before the swab has reached the proper location and is saturated with mucus. This technical lacuna may adversely affect the overall efficiency of NS-based sample collection.

During the SARS-CoV-2 surge in India, there were some innovations deployed by the Indian researchers in sample collection methods; the Center for Cellular and Molecular Biology (CCMB) developed a dry swab-based method for sample collection, which was approved by ICMR and the Drugs Controller General of India (DCGI) (45), this method does not require VTM to transport the sample. Another innovative method that found its relevance in SARS-CoV-2 was the Saline Gargle method developed by the Council of Scientific and Industrial Research - National Environmental Engineering Research Institute (CSIR-NEERI), which ICMR and DCGI approved (39). This method requires a 5 mL saline solution to be gargle-rinsed to wash out the virus into the saline solution; this saline solution is collected as a sample in a 10 mL container, which is then used for RNA release by using a TEP buffer.

### Sample transportation

Transportation of samples from testing centres to sequencing labs requires an efficient logistics network. The samples must be transported in a proper cold chain, the packaging should be strong enough to withstand wear and tear during the transport. Biosafety protocols should be followed at all levels while handling the samples. The samples must be transported to the sequencing labs with sample details. As per the guidelines of the Indian health ministry, the NS/OS VTM samples could be maintained at 4°C for <5 days, but if the samples are transported/stored exceeding 5 days, then it is required to maintain at -70°C (46). Maintaining the cold chain during transport in a vast country like India could be cumbersome, as there are various factors such as weather, road conditions, and transport availability which could lead to delays in sample transport or breaking of the cold chain. A decentralised WGS surveillance system could minimise the delay in transport and cold chain disruption. Therefore, it is better to adopt a decentralised strategy for an extensive and highly populated country like India.

### Inclusion of antigen positive samples in SARS-CoV-2 genomic surveillance

The distribution of samples is also a critical consideration in a genomic surveillance strategy; WHO, ECDC, and CDC recommend pursuing random and distributive sampling while selecting samples for genomic surveillance (22). In the case of an extensive and populous country like India, the COVID-19 testing strategy involved testing a large number of samples by antigen tests alongside the gold standard RT-PCR. However, the sequencing was done only for the positive samples by RT-PCR. The samples which tested positive by Antigen tests remained excluded from the sequencing pipeline. The Indian Council of Medical Research (ICMR) strategy considered antigen-positive cases true-positive without requiring a confirmatory RT-PCR.

In contrast, a confirmatory RT-PCR was later recommended for symptomatic cases which were antigen-negative. It is also important to note that almost 65% of the Indian population dwells in rural India (47). The SARS-Cov-2 testing in rural India largely depended on antigen testing due to the lack of available resources and logistics. A study has already validated the utility of sequencing SARS-CoV-2 from antigen test (48). We suggest that the antigen-tested SARS-CoV-2 positive samples can also be included in the SARS-CoV-2 genomic surveillance workflow (Figure 1).

### RNA extraction methods for SARS-CoV-2 testing and WGS

RNA extraction is an essential prerequisite for SARS-CoV-2 molecular testing and genome analysis. RNA extracts are subjected to a quantitative polymerase chain reaction (qPCR) for amplification-based detection of SARS-CoV-2 marker genes. The molecular detection of viruses is dependent on the quality of extracted RNA. It will not be out of place to consider this prerequisite step as a bottleneck; hence, it is necessary to optimise this step for further study. It is also pertinent to mention that the Ct value depends on not just the quantity of the target RNA but also the quality of the extracted RNA from the samples. Considering Ct value as a criterion for further selecting samples for WGS, the fate of samples selected for WGS depends on the preparatory step of RNA extraction. RNA extraction is a complex multi-step process, as RNA is a biomolecule; the contamination of RNAse in laboratories is very common, which causes RNA degradation in the laboratories while handling. The RNA extraction process could be carried out manually or by using an automatic RNA extractor system. Despite the availability of nucleic acid extraction kits and automation, there is still a need for a simple but efficient RNA extraction protocol that minimises steps and could also be scaled up easily to achieve high-throughput requirements. Briefly, a simple Proteinase K-based method of RNA extraction was evaluated by Ñique and colleagues (49) in which the samples were subjected to Proteinase K treatment (3 μg/μL) at 56°C for 10 min, followed by exposure to 98°C for 5 min and cooling at 4°C for 2 min.

The dry swab (45), and saline gargle (38) methods also bypass the multiple RNA extraction steps by using a Proteinase-K-based one-step extraction method, in which the samples are incubated with Tris EDTA Proteinase-K (TEP) buffer for 30 min at room temperature and then heated at 98°C for 6 min. This simple step provides reasonably good RNA suitable for PCR and WGS. The results of these one-step extraction methods were congruent with the results of the multi-step kit-based extraction method.

As a pilot study, CSIR-NEERI also conducted SARS-CoV-2 genomic surveillance using saline gargle samples in association with Nagpur Municipal Corporation for 500 SARS-CoV-2 positive cases (50). The saline gargle-based genomic surveillance was well received by the public at large, as it was a patient-friendly non-invasive method that attracted appreciable public participation as compared to VTM swab-based genomic surveillance. Samples collected through the gargle method contain a considerable amount of saliva; studies showed that the SARS-CoV-2 WGS results from saliva showed better sequencing depth than oropharyngeal samples (51).

Such a strategy can be deployed when there is a lack of costly RNA extraction kits due to economic constraints or overwhelming HPR. Also, such a strategy may prove very useful in rural and remote settings of a populated country like India.

### Importance of RTPCR pre-screening before SARS-CoV-2 WGS

Considering the constraints and non-uniformity of logistics, transportation and infrastructure available at various primary sample collection sites, which send the samples to INSACOG genome sequencing laboratories (IGSLs) through sentinel centres; we suggest not relying blindly on the RT-PCR Ct values as claimed by the RTPCR testing labs. It is worthy of performing a screening RT PCR of the samples before WGS. This RT PCR screening step will prevent a considerable number of sequencing failures due to RNA degradation in the received samples and also reduce the costly sequencing reagent loss.

### Utility of Oxford Nanopore Technology in the Indian context

Oxford nanopore technology is a next-generation sequencing (NGS) that uses an array of nano-scale pores (nanopores) over a membrane. The membrane is also electrically polarised when the genetic material passes through these nanopores at the rate of 450 bases per second (52) which generates a fluctuation in the membrane’s polarity. This fluctuation is characteristic of nucleotides passing through the nanopore. Every five nucleotides (A, T, G, C, and U) have corresponding electrical signals. A sensor records these signals, and a computer algorithm does the base calling. The first sequencer using this technology was commercially available in 2015. ONT has already been validated for its utility in genomic surveillance of SARS-CoV-2 in clinical samples (53) and wastewater samples (54). The advantage of this technology lies in its portability, simplicity, cost-effectiveness, and precision, which makes these devices an ideal choice for field deployment in settings like rural and remote areas of a populous nation like India. ONT-based sequencing technology can potentially be a primary technology for genomic surveillance in developing countries like India. Economic factors could be limiting when allocating resources for WGS surveillance in developing countries like India. The government may not prioritise WGS genomic surveillance over the financial allocations required for primary healthcare infrastructure. Studies have shown a direct correlation between GDP *per capita* and sequencing rates (55). We believe interventions like the deployment of mobile genomic surveillance units equipped with ONT sequencers can help achieve the goals of a decentralised facility for more comprehensive geographical coverage and proper representation of the population. Decentralising sequencing infrastructure will also improve efficiency by reducing delays in transport and sample spoilage due to logistic constraints. The development of such decentralised surveillance infrastructure will not only help deal with the SARS-CoV-2 pandemic but will also help serve as a ready platform for meeting the eventuality of future outbreaks.

## Comparing different SARS-CoV-2 genomic sequencing techniques

Next,-Generation Sequencing (NGS) techniques are being widely deployed for SARS-CoV-2 whole-genome sequencing. A study has evaluated the efficacy of four NGS-based SARS-CoV-2 WGS techniques (56). By using 24 clinical respiratory samples, the Ct value range of samples is (from 10.7 to 33.9). Four different strategies were employed for sequencing these samples. Out of which three were based upon Illumina sequencing (57): these include a customised metagenomic NGS (mNGS) protocol (58), two newly released kits, including an Illumina-developed hybridisation capture technique (DNA Prep with Enrichment kit, and Respiratory Virus Oligo Panel, RVOP) (59), and a Paragon Genomics-developed amplicon sequencing technique (CleanPlex SARS-CoV-2 kit) (60). This study has also assessed the widely used Oxford Nanopore Technologies (ONT) (61) sequencing method paired with the ARTIC Network’s amplicon sequencing technique (62). For eight samples with high viral loads, all four approaches produced almost entire genomes (>99%), with mNGS and RVOP generating the complete genomes. Amplicon-based enrichment techniques resulted in genome coverage >99% for all samples with moderate virus loads (Ct 20–25), but only 1 out of 8 samples sequenced with RVOP and 2 out of 8 samples sequenced with mNGS had genome coverage over 95%. Amplicon-based enrichment approaches were the most sensitive for low virus loads (Ct ≥ 25). Regarding identity in the entire consensus sequence, results from all approaches showed convergence. Due to the customised bioinformatics process setting a high threshold to call single nucleotide polymorphism (SNP) compared to reference sequence, CleanPlex only detected one mismatch in every three samples compared to the other approaches. A recently discovered 34 nt-deletion in ORF6 (63) was recognised adequately by all approaches; however, RVOP needed particular bioinformatic validation. It is evident from these studies that there is scope for increasing the sensitivity of WGS for low viral load (High Ct) samples. CleanPlex and ARTIC-ONT turned out to be the most sensitive method in this study, which could sequence the samples with a Ct value up to 33.9. In conclusion, mNGS continues to be the gold standard for samples with high viral loads to get the most information possible without bias; amplicon approaches like RVOP also result in very high coverage for low (Ct < 20) and mid (Ct ≤ 20 to <25). Amplicon-based enrichment is a suitable alternative for samples with higher Ct values (Ct > 25), especially the ARTIC-ONT technique, which did not exhibit any significant dropout problems. These highly sensitive NGS techniques will be helpful for genomic surveillance, especially in LPR-MPR scenarios, by including even weak positive samples with high Ct values.

## Discussion

The review article discusses the lacunas in the SARS-CoV-2 genomic surveillance and policy in the Indian scenario. It is not unreasonable to state that for an extensive and populous country like India, the dynamics of Infectious diseases are underplaying differently. There is an urgent need to revise the existing SARS-CoV-2 genomic surveillance strategy and policy so that the efforts made in the SARS-CoV-2 genomic surveillance are meaningful and effective. Various interventions are recommended in the review article, starting from the sample collection to the variant reporting system by WGS. Adaptation of decentralized system for SARS-CoV-2 genomic surveillance will contribute in rapid reporting of WGS data. This model will also ensure the better coverage of sequencing even to the remote areas of the country. The emergent pandemic wave driven by XBB and BF.7 variants has already made policymakers in India to think in the direction of reviewing the criteria of CT value for the selection of samples for WGS. We also observe the need to educate healthcare workers and policy makers at every level about the utility of genomic surviellance in effective management of the pandemic. We believe that if these recommendations are implemented, the COVID-19 mitigation policy of India may yield reasonably acceptable outcomes which will be in the interest of the public.

## Author contributions

ST and KK developed the concept for this review article and wrote and edited the manuscript draft. All authors contributed to the article and approved the submitted version.

## Funding

The research work was funded under a Major Lab Project (MLP) project number MLP-205, for the financial year 2022-2023. The junior research fellowship (JRF) for author ST was supported by CSIR.

## Conflict of interest

The authors declare that the research was conducted in the absence of any commercial or financial relationships that could be construed as a potential conflict of interest.

## Publisher’s note

All claims expressed in this article are solely those of the authors and do not necessarily represent those of their affiliated organizations, or those of the publisher, the editors and the reviewers. Any product that may be evaluated in this article, or claim that may be made by its manufacturer, is not guaranteed or endorsed by the publisher.
